# The biochemical and molecular mechanisms of plants: a review on insect herbivory

**DOI:** 10.1080/15592324.2024.2439248

**Published:** 2024-12-26

**Authors:** Afeez Adesina Adedayo, Richard Musser, Mari Aanaenson, Olubukola Oluranti Babalola

**Affiliations:** a Department of Biological Sciences, Western Illinois University, 1 University Circle, Macomb, IL, USA; b Food Security and Safety Focus Area, Faculty of Natural and Agricultural Sciences, North-West University, Mmabatho, South Africa; c Department of Life Sciences, Imperial College London, Ascot, Berkshire, UK

**Keywords:** Elicitor, insect detoxification, plant defense, plant signaling, phytopathogens

## Abstract

Biochemical and molecular mechanisms have been essential mechanisms to reduce various insect attacks on plants. The biochemical methods are wide involving direct and indirect defenses. The defensive chemical substances are secreted effectively to the wound caused by the herbivores (insects and phytopathogens) on plants. Plants responded by producing VOCs which draw the natural enemies of the insects and phytopathogens. The progress observed in the cognition of the stimulus in plants and their potential to control the responses is characterized by the modification observed in molecular mechanisms which shifts our attention to the development of the endogenous resistance methods of preserving crops. The main objective of implementing a biotechnological mechanism in crop production is to employ durable and multimechanistic alternatives to insect pests via the stimulus the plant produces upon encountering the insect attack.

## 1. Introduction

The global population will attain 10 billion according to the estimation made as a result of the increase in projection in the next four decades.^1^ The sole importance of agricultural practice is to generate abundant production of crops and food materials that are environmentally stable but to our surprise, insects have contributed by reducing the production of crops by 10–20%.^2,3^ However, genetically modified (GM) technology has helped in the production of crops that possess endogenous resistance to the activities possessed by insect pests.^4–6^ Some studies have reported how the genetic modification has been effective in various crops including *Zea mays*,^7^ *Gossypium hirsutum*,^8^ *Solanum lycopersicum*^9^, *Vigna unguiculata*,^10^ and *Solanum tuberosum*,^11^ etc. which reveals various genes coding for entomocidal δ-endotoxin produced by *Bacillus thuringiensis*.^12,13^ These bacteria have been employed in commercial farming of more than 10 million hectares all around the world that stimulate the production of toxic chemicals against the invasion of insects including moths, beetles, aphids, butterflies, etc.^14,15^ The toxins produced by the *Bacillus thuringiensis* are likewise effective on insect products thereby conferring protection to crops for abundant production and improving agricultural sustainability.^16^ Despite the potential of these microbes toward insect invasion of crops, further methods of creating resistance against the insects should be produced as alternatives to the use of molecular techniques which this review discussed some of the methods. Various studies have reported some vital aspects relating to the interaction of plants and insects.^17–19^ However, ecologists have explained the functional complexity of how plants resist insect invasion via molecular methods. The introduction of high-throughput techniques employed in plant-phytopathogen interactions may not be truly employed in the study of the interaction between plants and insects.

The molecular aspects involved the use of tools to show the essential processes mediating direct and indirect defenses, yet, little was known about the application of molecular methods. The application of DNA microarrays and Polymerase Chain Reaction (PCR) has availed the transcriptional changes that take place once there is an attack on plants by insects triggering plant host response.^20^ Moreover, this review focused on various methods that can be used to determine the plant-insect interaction involving defense responses.

## 2. Herbivory

Plants do stimulate response toward the invasion of insect attack through biochemical, structural, and molecular methods.^21^ The process by which plants are been fed upon by insect (herbivore) attack is known as herbivory.^22^ The effectual biochemical methods against the herbivores can be via direct or indirect defense mechanisms.^23^ These defense mechanisms can be stimulated either constitutively or effectively to plant destruction thereby inhibiting the herbivore survival and development. As a result, plants produce chemical substances called volatile organic compounds (VOCs) that invite microbial communities^24^ that antagonize the invasion of herbivores (insect pests) by the process referred to as induced systemic resistance. This process is the essential biological process of controlling pest in agriculture which do not rule out insect reduction.

Several years ago, various progressions were witnessed while investigating plants’ induced responses against stresses. Despite induced response carries out metabolic necessities that are essential in reducing the stresses caused by VOCs in response to herbivore aggression. Changes obtained in defensive materials produced by plants triggered by herbivore invasion produce the effectual means of the VOCs during the plant-insect interaction which controls the herbivores’ behaviors.^25^ Early production of induced response provides advantages to plants and inhibits the existence of either phytopathogens or herbivore invasion,^26^ unlike plant fitness. Improvement in plant-insect interaction has strengthened the knowledge of defense evolution triggered by plants against insects.

## 3. Direct defenses

**Figure 1.**
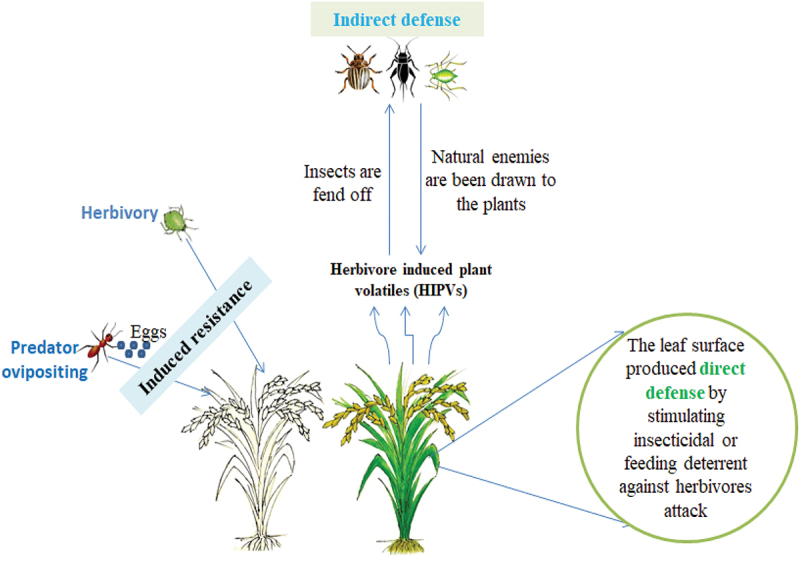
Direct and indirect defense mechanism of plant against insect attack.

This reveals the plant traits including primary and secondary metabolites, trichomes, thorns, etc. that affect insect activity and are classified according to their potential.^27^ Typical examples are polyphenol and antinutritive proteins that induce mechanical damage in plants. They are also known as protease inhibitors (PI) responsible for reducing elastases in the midgut of a larva as observed in Figure 1.

### 3.1. Plant morphology

The structure of the plant is the first defense against herbivory and carries out essential functions in plant resistance to herbivore attack.^28^ This first line of defense in plants contains physical obstacles via the organization of waxy cuticles or outgrowing of trichomes, spines, setae, etc. Anatomical and structural traits are structural defenses that bestow the healthy status of the plant through the rate of reducing herbivore nutrition as well as the projection observed on the cell wall rigidity due to suberization and lignification.^29^ The thorns, spines, and trichomes are involved in plant protection against insect attacks by reducing their potential to digest the plant tissue which may have caused damage to plants.^30^

### 3.2. Trichomes

They are involved in plant defense against herbivores and carry out deterrent and toxic effects. The density of trichomes turned out to inhibit insect reproduction, larval or insect nutrition, incapacitate insects and other groups of arthropods’ movement on the plants which safeguard the plant leaves from the herbivore. Trichomes can exist in various forms including branched, unbranched, spiral, hooked, straight, glandular, or non glandular. Glandular types produce secondary metabolites like terpenoids, alkaloids, and flavonoids. These chemicals act as repellents to the insects because they are poisonous to the insects and phytopathogens, so they are known to carry out chemical and structural defense mechanisms. Some studies have reported the effect of trichomes protecting plants by producing chemical substances in response to the insect invasion as observed in Table 1. Differences are observed in the density of trichomes within the weeks of insect attack. This is also noticed by inducing stimulus against the insect in glandular or non-glandular trichomes.

**Table 1.** Plants produce trichomes or secondary metabolites to inhibit the effects of insects and phytopathogens.

Insect species/Spoilage organisms	Plant(s)	Trichomes products/Secondary metabolites	Activities	References
Grapevine moths, mealybugs, phylloxera, and spotted lanternfly, etc.	*Vitis vinifera*	Primary/secondary, metabolites, elicitors, volatile organic chemicals (VOCs)	The chemical produced acts as a defense mechanism against insect attack	31
*Macrosiphum euphorbiae*, *Spodoptera littoralis* caterpillar	*Solanum lycopersicum*	Jasmonic acid (JA)	Tomato plants produce glandular trichomes that confer defense against herbivores	32
*Trichoplusia ni*	*Solanum lycopersicum* mutant	*Odorless-2* and *jasmonic acid–insensitive1*	The study reveals how plant genotype contributes to the digestion of lipid metabolism	33
*Myzus persicae*	*Nicotiana benthamiana, Arabidopsis thaliana*	Camphene, myrcene, and limonene	The secondary metabolite produced performs an important function in plant defense mechanism	34
*Cnaphalocrocis medinalis*	*Oryza sativa*	Jasmonate, salicylic acid, and flavonoids	The secondary metabolites are produced as a chemical defense mechanism against herbivore attack	35
*Bemisia tabaci*	*Glycine max*	Trichome length, angle, and density on the plant leaves	The foliar trichomes were characterized by producing genes responsible for producing chemical material that reduced the population of the white flies	36
Insect pests, phytopathogens	Plant species	Alkaloid, terpenoid, glycoside, and phenolic compound	The secondary metabolites prevent plants from biotic stressors by attracting microbial antagonists that can prevent the insects, repelling them insect from attacking the plants or creating toxic effects on the plants	37
*Bemisia tabaci*	Plant species	Terpenoid, jasmonate	The chemical substance confers resistance against the insects	38
*Aceria litchii*	*Litchi chinensis*	Trichome density	The effect of temperature and trichome density reduces the potential and population of mites	39
*Bemisia tabaci*	*Solanum lycopersicum*	Acylsugar-associated type IV	The trichomes produce the substances to control the white flies	40
–	*Artemisia annua*	Alkaloids, terpenoids, and other organic substances	The glandular trichome is important for conveying cutin and wax materials	41
–	Plant species	Defensive proteins, phenolics, terpenoids, methyl ketone O-acyl sugars	These metabolites contribute to plant growth promotion and are likewise used in therapeutic and cosmetic industries	42
*Phthorimaea absoluta*	*Solanum lycopersicum*	Acylsugar production by trichome	It reduces the insect population by forming resistance	43
Arthropods	Cereals (*Oryza sativa, Triticum aestivum, Sorghum bicolor, Zea mays*)	Jasmonic acid and salicylic acid	The chemical materials participate in plant signaling defense mechanisms.	44

### 3.3. Secondary metabolites

These are compounds produced by plant tissues that do not contribute to the growth and development of plants but act as a medium for preventing plants from harm (defense mechanism) as observed in Table 1. These chemicals produced can be constitutive (phytoanticipins) that are triggered by β-glucosidase to produce some biocidal aglycone compounds or induced (phytoalexins) forms that are stimulated when phytopathogens or insects launch attacks on the plants.^45^ A typical example of phytoanticipins include glucosinolates that myrosinases hydrolyze in the disruption of plant tissues, other examples are Benzoxazinoids that are widely spread among the Poaceae.^46^ During the process of defense in plants, there is hydrolyzation of Benzoxazinoids-glycosides with the aid of β-glycosidases while the tissues are been damaged to produce biocidal aglycone which is an essential function of plant defense against insects. A typical example of phytoalexins is alkaloids, isoflavonoids, terpenoids, etc. that control the survival and functions of insects.^37^ These metabolites improve plant fitness aside from their potential to prevent biotic stress caused by herbivores. Despite secondary metabolites being known for their defensive potentials, emerging signaling pathways are also required to be conducted. Profiling the secondary metabolites, mass spectrometry has been employed in the analysis as well as the expression of genes.^47^ So, the profiling of the secondary metabolites amounts to the determination of new signaling molecules used as plant resistance against insects.

#### 3.3.1. Phenolics

This is among the constituents of the secondary metabolites containing one of the most usual and distributed defensive mechanisms that carry out the function of preventing herbivore attacks. Phenols have been reported to act against insects and also repel invading phytopathogens and other plants involved in the competition.^48–50^ Lignin is a phenolic heteropolymer that carries out significant functions in the process of plant defense. The heteropolymer reduces the number of phytopathogens by toughing the leaf membrane and this prevents herbivores from causing damage to the leaves but due to the hardness of the leaf’s surface, it limits the nutrient value of such leaves.^30^ The process of manufacturing lignin stimulated via phytopathogens or herbivore attacks inhibits the growth of such herbivore fertility. When polyphenol is oxidized by the action of peroxidase or polyphenol oxidase, it leads to plant defense potential against insects.^51^ Once the oxidation process is complete, quinone is produced as the product of phenols which attach to the proteins found on the leaves covalently and therefore reduce the digestive rate of the proteins in the insects. Quinones are likewise toxic to insects thereby reducing insect attacks on plants.^52^ The nutritional advantage of protein in plants has been reduced by the alkylation of the amino acids that turn out negatively on the development of insects. Phenols carry out significant functions in the cyclic reduction of reactive oxygen species (ROS) which include hydroxide radicals, and superoxide anions that trigger reactions resulting in the production of defensive enzymes.^53^

#### 3.3.2. Flavonoids

This chemical substance is another secondary metabolite produced by plants. It acts as a central function in every area of plant life, especially plant-soil microbial interaction.^54^ The metabolite confers protection on plants against biotic (phytopathogens and herbivores) and abiotic (extreme temperature, salinity, pH, radiations, etc.) stresses.^46^ Flavonoids are known to be cytotoxic and can associate with various enzymes via the process of complexation. Flavonoids can act on free radicals like ROS and inhibit their production by chelating metals in the soil.^55^ They are of various types according to their function and they are; flavonols, aurones, flavan, flavones, anthocyanins, flavanones, dihydroflavonols, proanthocyanidins, and chalcones.

#### 3.3.3. Tannins

This is another secondary metabolite that possessed a strong deleterious effect on insects and phytopathogens by inhibiting their growth via reduction of nutrient uptake, attaching to the insect proteins, and midgut lesions in the insects. Tannins are referred to as bitter polyphenols and as nutrition hindrances to various herbivores.^56^ This chemical can precipitate protein through covalent or hydrogen bonding of proteins – NH_2_ groups.^57^ They can likewise chelate metallic ions and decrease the availability to the insects.^56^ When insects take in the tannins, it decreases the rate of protein digestion thereby reducing the nutritive content of the plants to the insects.^58^ The main function of tannins is to protect plants against biotic and abiotic stresses as well as stimulate systemic resistance against phytopathogens that can attack the plants causing deterioration of their health status.^59^ Tannins do occur in concentrated (oligomeric) or polymeric flavonoids which are referred to as proanthocyanidins.^56^ They possess various shapes and roles they carry out. They are regarded as nutritive impediments against insects like brown-tail moth, spongy moth, winter moth, etc. Certain genes are accountable for the tannins’ production upon herbivore attack. Moreover, the stimulation of tannins is triggered by light stress or subjected to UV light.^60^

### 3.4. Plant defensive proteins

The association between plants and insects is significant for their survival. Healthy plants are been attracted to insects in other to feed on as well as to serve as a medium for them to mate for the production of young ones alive. The nutrients required by insects are the same as the required nutrients for other animals and the instability of the digestive and absorption of proteins leads to consequences in insect physiology. Changes that occur in gene expression in stress conditions constitute insect attacks that result in protein changes qualitatively and quantitatively that carry out significant functions in oxidative and signal transduction defense.^48^ In the midgut of an insect, the proteins assimilated are stable and are transported through the gut wall to hemolymph. Changes observed in the structure of amino acids of the protein sequence contribute to the role of such protein. Another procedure involved breaking down of protein which is an anti-insect activity that can promote the production of protease inhibitors that disallow proteolysis of toxic proteins and therefore permit them to carry out their defensive activity.

### 3.5. Enzymes

Host plant resistance is one of the characteristics of plants to secure itself from the attack of insects and other phytopathogens and this is achieved via the production of various enzymes including peroxidase, ascorbate peroxidases (PODs), polyphenol oxidases (PPOs),^61^ etc through oxidation of mono or dihydroxy phenols resulting to the production of reactive o-quinones, that are polymerized with nucleophilic groups of proteins as a result of electrophilic nature. The following are examples of antioxidative enzymes; superoxide dismutase, lipoxygenase, phenylalanine ammonia lyase, etc.

## 4. Indirect defenses

This is a type of plant defense mechanism that attracts plant parasites or predators to be involved in a specific defensive role against insect attack. This type of defensive mechanism can be induced due to the potential of damage caused and elicitors produced by the insects.^62^ Secretion of volatile organic chemicals like extrafloral nectar (EFN) introduces an association between predators and plants that can decrease the population of insects.^63^ Indirect defense stimuli in Figure 1 have obtained improved courtesy and have a better understanding of ecological, biochemical, and physiological stages.^64^

### 4.1. Production of plant volatiles by herbivores

The indirect way by which plants protect themselves from herbivore attack is by producing nonvolatile and a blend of volatile substances. Herbivore-induced plant volatiles (HIPVs) carry out significant functions in plant defense via attraction to natural predators of the herbivores or by avoiding the insects being able to multiply by laying eggs.^65,66^ This chemical substance is a lipophilic substance that possesses extreme vapor pressure which is produced by the leaves, fruits, flowers, and roots in effect to insect attacks. The volatile chemicals produced in response to insect attacks are special for plant-insect interactions constituting the plants and natural enemies.^22^ The HIPVs arbitrate the association occurring between plants and species of arthropods or phytopathogens causing damage to plants in Table 2.

**Table 2.** Phytopathogens and arthropods and their effects on the plants.

Phytopathogens	Arthropods	Plant-herbivore interaction	References
*Fusarium*	Insects	*Fusarium* and insects involved in symbiosis association in plant disease epidemiology	67
*Gluconobacter oxydans* and *Acetobacter acetic*	*Drosophila melanogaster* and *D. suzukii*	The coexistence of the insect vectors promotes the emergence of phytopathogens in the wounded grape plants	68
*Stemphylium* sp.	*Nezara viridula* and its egg parasitoid (*Trissolcus basalis*)	*Trissolcus basalis* utilizes oviposition-induced plant volatiles	69
*Pseudomonas syringae*	*Tetranychus urticae*	The association of the bacteria with the insect compromised the insect’s attack on the plants	70
Gut bacteria (*Citrobacter* spp.)	*Bactrocera dorsalis*	These bacteria in the gut of insects give them the potential to feed and reproduce on the affected plants	71
yeast-like symbionts (YLSs)	*Nilaparvata lugens*	The microbes provide essential amino acids and sterol precursors to assist in recycling nitrogen	72
*Fusarium* spp.	*Xyleborus affinis*	The fungi and insects induce damage to the plant host	73
*Phytophthora infestans, Xanthomonas campestris, Puccinia graminis*	Arthropod pests	The phytopathogens and the insect pests destroy crops including; bananas and wheat	74
*Geminiviridae, Closteroviridae, Nanoviridae, Rhabdoviridae*, and *Potyviridae*	Aphididae, *Bemisia tabaci*	The association between the insect and the virus improves pathogen transmission thereby encouraging the destruction of plants	75
*Botrytis cinerea, Fusarium oxysporum*, and *Alternaria alternate*	*Chrysodeixis chalcites*	The insect species and the phytopathogens attack and reduce the production of *Magonia pubescens*	76
*Xanthomonas citri, Ralstonia solanacearum, Alternaria alternata, Aspergillus niger, Botrytis cinerea, Colletotrichum graminicola, Fusarium oxysporum, F. verticillioides, Pythium ultimum, Verticillium dahliae, V. longisporum*, and *Urocystis agropyri*	*Aphis gossypii, Diaphorina citri, Schizaphis graminum, Tricoplusia ni, Plutella xylostella, Spodoptera frugiperda*, and *Leptinotarsa decemlineata*	They are pathogenic organisms and insect pests attacking plants	77
Nematodes and protozoa	Fleas, lice, flies, mites, and ticks	These involve crop and animal pests that affect the growth of crop plants	78

Based on the type of insect feeding, various routes of defense signaling are initiated, which stimulate particular volatile compounds including green leafy volatiles (GLVs), terpenes, methyl salicylate, ethylene, etc.^22^ Out of these metabolites, GLVs are highly reactive electrophiles that take part in stress and defense signals.^79^ This metabolite is involved in a significant function in plant defense by drawing natural enemies. Methyl salicylate (MeSA) is the most often substance in HIPVs and is regarded as the headspace of plants attached by insects. MeSA is a cosmopolitan material produced by plant leaves and other floral parts which attract insects and other predators.^80^ The production of VOCs is regarded as an excellent response to herbivore attacks. However, the HIPVs safeguard the plants by a direct method involving repulsion, toxicity, and deterring the herbivore^22^ or by an indirect method involving attraction of natural enemies of the insect and phytopathogens, thereby conferring protection to the plant from herbivore attack.^62^ However, Terpenoid, lipoxygenase, and Shikimic acid carry out specific roles in the defense mechanisms of plants directly or indirectly.

Plants’ aerial parts like roots have also been reported to produce VOCs to defend plants within the rhizosphere soil.^26,81^ The VOCs also affect the root-feeding insect pests. Some insects feeding on plant roots activate the production of 1,8-cineole also known as monoterpene that repels and is toxic to insects. This volatile substance also reduces the development of *Brassica campestris* seedlings as a result of a reduction of cell multiplication that occurs rapidly compared to cell elongation because both cell multiplication and elongation occur in the plant roots.^82^

### 4.2. Insect defense elicitors

The metabolites, transcriptome, and proteome of plants can be altered when insects stimulate physical and chemical factors as observed in secretions produced by insects orally or substances produced during the process of oviposition.^83^ Insects can stimulate responses in plants through the body fluids and regurgitate produced by the insects.^84^ The elicitors produced defense deviated based on the elicitor forms and bioprocess required. The significant substances produced in the oral secretion of insects are fatty acid-amino conjugates (FACs).^85^ In the oral secretion (OS) of insects (armyworm larvae) are found *N*-(17-hydroxylinolenoyl)-L-glutamine (Volicitin) and volicition. When volicitin is employed on maize plants, it stimulates the production of elicitors that draw natural enemies of the larvae feeding on the plant.^86^ The FACs is a chemical substance produced by insects that can trigger the pathway of mitogen-activated protein kinase (MAPK) that is involved in the control of growth and development in plants and significant function in signaling transduction while responding to both biotic (phytopathogens and insect attack) and abiotic stresses (ROS, heat, cold, salinity, UV, pH, drought, etc.).

### 4.3. Phytohormones and their functions

The plants can protect themselves from herbivore attacks through signal transduction pathways that are regulated by the potential of the Phytohormone. Phytohormones are chemical substances produced by plant cells that carry out significant functions in controlling plant development and disease-resistant mechanisms caused by phytopathogens^87^ or through wounds invented by insect attacks.^88^ Plant(s) can be involved in intra or inter-association with one another. Some plant hormones are required during this interaction to prevent the destruction that can be caused by herbivores. The defense mechanisms in plants are mediated by signal transduction pathways employing some phytohormones including ethylene (ET), jasmonic acid (JA), salicylic acid (SA), auxin (indole acetic acid (IAA)), etc.^89^ With the aid of these hormones, plant genes are activated which are duly responsible for certain functions upon attack or wound caused by herbivores and work as biocontrol or synergistically against the phytopathogens and the insects.

#### 4.3.1. Jasmonate

Jasmonate is the bioactive derivative in the pathway of biosynthesis of jasmonic acid (JA). JA is one of the essential phytohormones used by plants to defend themselves against herbivore attacks and also initiate direct and indirect defense mechanisms.^90^ JA is produced from linolenic acid via the octadecanoid pathway and aggregates after herbivore attack. The defense mechanism produced by jasmonate constitutes alkaloid secretion, antioxidative enzymes, VOCs, trichome production, etc.^91^ JA is known to regulate the plant genes involved in defense mechanisms. The amount of indole glucosinolate present are essential defensive material stimulated by jasmonates. Jasmonates are also associated with the COI1 of ubiquitin (E3) ligase complex thereby improving the attachment of COI1 to jasmonate ZIm-domain proteins leading to lysis of JAZ proteins that inhibit the expression of genes of JA-inducible.^92^ JA demonstrates its effect on calcium-dependent protein kinase (CDPK) copy and its potential in potato plants. To control biotic and abiotic stresses in plants,^93^ JA has demonstrated great potential to reduce these effects via signal transduction. Moreover, JA also contributes specific functions in direct and indirect resistance against insects via the stimulation of defensive substances.^23^ A typical example has been observed in the production of extrafloral nectar (EFN) by JA which is utilized by natural enemies of the insect. JA likewise produces POD and PPO as reported by Waheed, Haxim, Kahar, Islam, Ahmad, Khan, Ghramh, Alqahtani, Hashemand and Daoyuan.^94^

#### 4.3.2. Salicylic acid

This is a benzoic acid derivative that takes part in plant defense mechanisms. This phytohormone is endogenous and necessary for controlling plant growth and produces metabolic and physiological responses in the plant’s defense to improve the growth of plants.^95^ The effect of SA depends on the regulatory protein known as pathogenesis-related gene 1 (NPR1) expression. The initiation of the NPR1 gene via redox reaction by collection of SA and its translocation to the nucleus binds not directly to the nucleus, but instead functions via transcription factors.^96^ The defense mechanism against types of insects including piercing and sucking insects compared to biting and chewing insects is being stimulated by SA.^1^ The signaling molecule of SA contributes to local defense and likewise induces systemic resistance in plants. In the SA pathway, the stimulation of ROS leads to the production of systemic resistance against herbivores by plants as observed in tomato plants against powdery mildew disease as reported by.^97^ SA involves in defending plants against piercing and sucking insects (aphids) compared to biting and chewing insects (ants). SA signaling molecules also contribute to the defense of plants and likewise induce systemic resistance. The initiation of ROS through the pathway of SA has been confirmed to stimulate resistance against insect pests in plants.^98^ SA can produce H_2_O_2_ in plants as a result of the potential to destroy the insect digestive system which results in the reduction of insects attacking the plants.^21^ Moreover, SA can produce volatiles during signaling that draw the natural enemies of the insects.

#### 4.3.3. Ethylene (ET)

This is another significant plant hormone that performs an essential function in plant defense against arthropods. The pathway of ethylene signaling carries out an essential function in stimulating plant defense against arthropods and phytopathogens either through direct or indirect mechanisms. Nevertheless, the pathway of ET signaling functions antagonistically or synergistically together with the expression of JA in plant defense against some insects and phytopathogens.^99^ Both JA and ET have been reported in some studies to work together in some plants to inhibit disease invasion as well as insect attacks.^100,101^ The precursor or substance relating to ET is 1-aminocyclopropane-1-carboxylic acid has revealed how to promote the production of JA from treated detached leaves.^102^ Other substances including (Z)-3-hexen-ol, volicitin, etc can be produced by ethylene in maize plants.

### 4.4. Calcium ions (Ca^2+^) potential in plant defense

In the herbivory process, defense elicitors in plants carry out various signal transduction pathways. Ca^2+^ signaling is an immediate reaction that takes place in insect-plant interaction, where Ca^2+^ is regarded as the second messenger that results in several signaling pathways in plants.^25^ When an insect triggers signals, the signals are distributed in the leaves which results in depolarization of the cells in the leaf through Ca^2+^ ion-dependent transmembrane potential (Vm) occurrence at the site of the wound. Furthermore, the cell undergoes hyperpolarization when the Ca^2+^ leaves the surrounding cells of the leaves.^103^ The cell organelles and apoplastic substance constitute a high concentration of Ca^2+^ compared to the cytoplasm found in the inner part of the cell. However, when an insect launches an attack, the Ca^2+^ concentration in the cytoplasm increases thereby activating calmodulin, the calcium-sensing protein which in turn attaches to calcium-dependent protein kinase (CDPKs) to improve the process of transcriptional change and phosphorylation. CDPKs were known for their main function against both biotic and abiotic stresses.^104^ They produce Ca^2+^ sensors that are made up of protein kinase and calmodulin in a unit of a polypeptide.^105,106^

### 4.5. Reactive oxygen species (ROS)

Plant oxidative state is a significant method that encourages plants to protect themselves from stresses. The inauguration of ROS in a rapid state is regarded as a frequent process in plant oxidative stress because of biotic and abiotic factors. ROS perform various signaling roles that arbitrate multiple responses and likewise function as specific toxins.^107^ To respond to various biotic stresses, ROS are typically produced and this response is problematic. ROS constitute superoxide-like hydroxyl radicals (HO-), and hydrogen peroxide (H_2_O_2_).^108^ Specific signaling pathways are initiated through ROS of various kinds most importantly MAPKs.^109^ However, an increase in the content of ROS is known as an oxidative burst. Once an insect has inflicted a wound on the plant, there is an aggregation of ROS in apoplastic and symplastic areas aside from their main amount in the exocellular matrix, plasma membrane, and mitochondria.^110^ When the plasma membrane (apoplastic) breaks, the ROS acts as the preliminary roadblock against another attack that may be incurred by the phytopathogens and the insects. So, ROS can fight against and cause huge damage to the nucleic acids, lipids, or proteins.

To prevent the ROS from fighting the host plant cell, the cell should be able to manufacture ROS-devouring systems to take away excess ROS in other to keep the normal ROS concentration. In all types of ROS, diffusible and high-stability H_2_O_2_ is regarded as the brain of induces systemic resistance in plants to various types of stresses.^111^ However, H_2_O_2_ can be manufactured in many ways. Yet, the oxidative burst can take place via NADPH complex membrane-bound activation. Superoxide anions are produced by NADPH oxidase from the cell membrane or extracellularly by apoplast which is further changed to H_2_O_2_ with the help of an enzyme superoxide dismutase (SOD).^112^ Apart from the direct effect occurring in insects and phytopathogens, H_2_O_2_ activates a net reaction that results in the defense gene expression that prevents the plant from other attacks from insects and phytopathogens.^48^ The introduction of H_2_O_2_ in *Arabidopsis* leads to up-or down-regulation of genes, proposing how ROS functions as a secondary messenger that regulates the expression of genes.^113^ ROS essential function in regulating the cross-linking of cell wall materials with the aid of peroxidase and also for the initiation of some genes involved in defense. Once there is an oxidative wound in the midgut of an insect, then oxidative changes occur as a result of H_2_O_2_ accumulation. In plants are some molecular and physiological changes that occur in plants against insect attack that are activated by H_2_O_2_, and its remains increase as long as the insect continues to attack.^114^ ROS regulates the activation of gene expression and makes available the required defenses by controlling the process of transcription^115^ or interaction with other signal materials including phosphorylation in plants while responding to various stresses encountered.

### 4.6. Transgenerational induced resistance (TIR) to insects

The potential of a plant to transmit immunity or resistance to diseases and insect invasion to its progeny or generation is known as transgenerational immunity.^116^ Plants undergo biotic and abiotic stresses that induce resistance in maternal plants and the generation produced. This maternal-induced resistance is called transgenerational immunity which was known to safeguard the offspring of the plants subjected to herbivory aside production of vigorous seeds.^117^ Few studies have been reported on transgenerational immunity that prevents herbivore attacks on plants. Kambona, Koua, Léon and Ballvora^118^ reported how *Raphanus raphanistrum* was attacked by *P. rapae* while Moreira, Abdala‐Roberts, Gols, Lago‐Núñez, Rasmann, Röder, Soengas, Vázquez‐González and Cartea^119^ reported how *R. raphanistrum* was treated with JA accompanied by an abundance of induced systemic resistance on the herbivore.^120^ Abiotic stresses including flood, heat, and cold in *Arabidopsis* plants lead to linkage of the same kind of frequency and increment in the methylation of the genome. It results in the stimulation of resistance to various stresses in the new generation produced. Maternal plants constituting low to medium degree of attacks conferred by the insects could generate vigorous seeds and insect-resistant seedlings. Moreover, other studies should be employed to comprehend the genetic and molecular methods in an association related to signaling.^121,122^ Plant signaling are essential for plant transgenerational immunity because they allow parents to communicate their stress responses to the generation produced by them.

**Figure 2.**
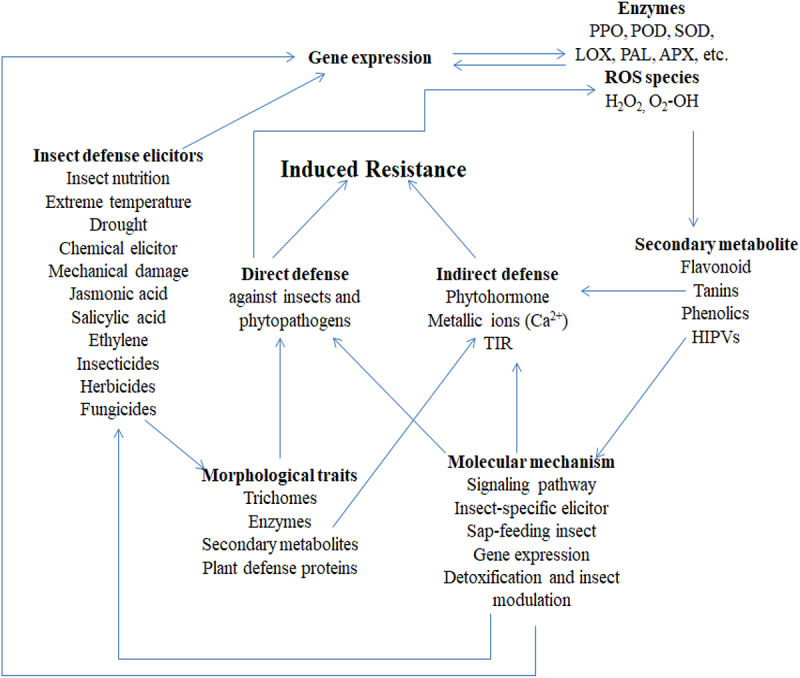
Mechanisms involved in plant defense.

Therefore, plant-insect interaction should not only be based on genetic methods but also involve the epigenetic control of the defense signaling pathway as revealed in Figure 2. The insect responses as a result of significant studies have been reported for mobile siRNA signals and methylation of DNA in changes observed during the gene expression. There are various mechanisms^123^ involved in this process is carried out as described below;

Epigenetic Changes: Due to pathogen or insect invasion exposure, parent plants may experience epigenetic changes, such as DNA methylation or histone modification. These alterations may be passed on, preparing the following generation’s immune system to react more quickly.

Signaling Molecules: Salicylic acid and other signaling molecules produced by infected plants can be transmitted to plant seeds. These chemicals help the progeny react better to phytopathogens and insect threats in the future.

Modified Metabolism: Stress on parents can alter a seed’s metabolic makeup, which may increase the generation’s ability to produce defense-related chemical substances.

Microbiome Influence: How plants’ offspring react to infections can be influenced by the microbial communities linked to the parent plant, which can also have an impact on seed immunity and health.

## 5. Molecular mechanisms

Researchers have comprehended how *Bacillus thuringiensis* (Bt) plants like Bt eggplant, Bt maize, and Bt cotton undergo multimechanistic resistance to insects. The molecular methods undergoing this elaborated effects (Figure 2). Nevertheless, reports have revealed how plant-insect-stimulated transcriptome employing microarray and differential methods have avail knowledge in plant-insect interaction.^83^ JA carries out its main function in response to insects. The pathway of JA is initiated by plants and SA is changed through methylation into volatile MeSA that draws natural enemies resulting in phytohormones’ synergistic effect in indirect defense.^124^

### 5.1. Signaling

**Figure 3.**
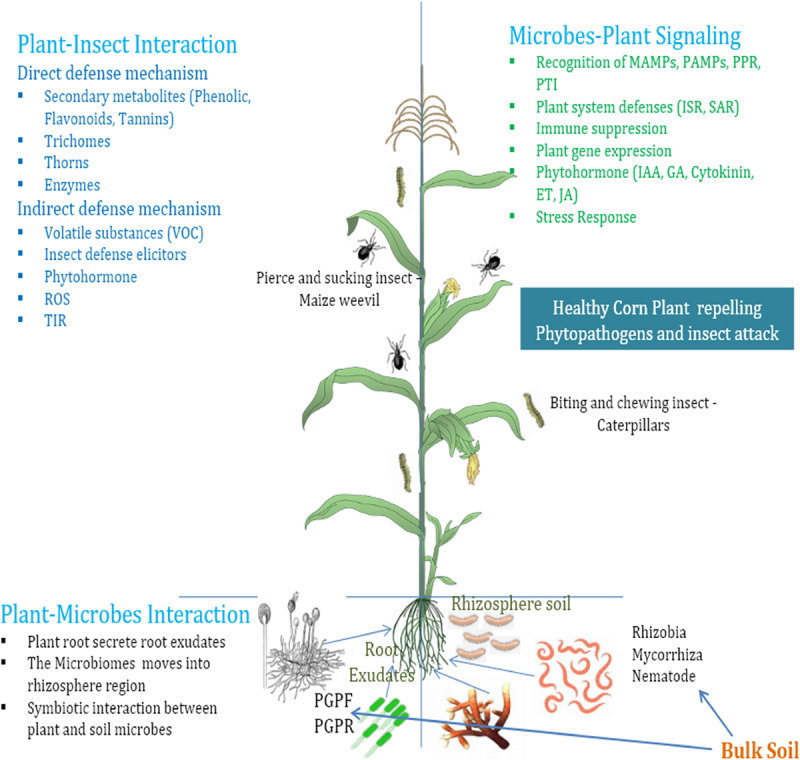
Various interaction involved by plant, microbes (beneficial and pathogenic), and insects.

This process performs the role of controlling insect-stimulated expression of genes and will yield some possibility to regulate the responses. JA, SA, and ET do not trigger defenses independently through a linear cascade of signaling to find out the particular responses (Figure 3). Direct cognition of the interaction can be utilized in transgenic plants containing phytopathogens and insect resistance.^74^

### 5.2. Insect-specific elicitors

The basic process utilized by plants to observe the proximity of insects has revealed the oral secretion of insects. Prajapati, Vijayan and Vadassery^125^ revealed responses were observed in tobacco wounds modified by Manduca sexta regurgitate to stimulate JA and ET production to known specific elicitors as fatty acid conjugates. The conjugates of fatty acid and volicitin obtained in the lepidopteran oral secretions lead to gene expression upregulation.^22^ Due to the high production of VOCs when added to maize plants, is an increment in the concentration of volicitin produced from beet armyworm larvae oral secretion,^126^ and this was known to be the initial insect-specific elicitor. The complete arrangement of volicitin containing hydroxylinolenoyl has been found and enantiomers removed.^126^

### 5.3. Sap-feeding insects

When an insect feeds on plants, it causes wounds on the plant tissues, mostly the content of the vascular bundles (xylem and phloem). When their proboscis is inserted into the tissue containing overlying cells, they cause damage to the cell and reduce the stimulation of wound response. In opposition to the damage effect on the plant tissues, plant responses are activated immediately after an insect attack with a typical example of interaction occurring between various genes.^127^

### 5.4. Insect response to plant defense

The utilization of plant protection methods against insects counts on insect or other phytopathogen response to chemical compounds secreted by the plant.^15^ Due to this, the herbivores can adapt to compounds produced by the plant to defend themselves. A common example is obtained in lepidopteran and coleopteran which can accommodate the production of proteinases and are unreactive to suppression. The utilization of plant protection methods is contingent on insect pests’ response to the chemical substances produced by plants against the insects.^128^ Due to coevolution, these insects adapted and tolerated plant insecticidal chemicals for defense. Through intricate signaling channels and reactions, interactions among plants, insects, and phytopathogens substantially impact gene expression.^129^ These common interactions that influence plant gene expression are phytopathogen-herbivores’ recognition and response, cross-talk between pathways, insect feeding and damage, epigenetic change, and metabolic changes

#### 5.4.1. Phytopathogen-herbivores’ recognition and response

Pattern Recognition Receptors (PRRs) – Plant cells use Pattern Recognition Receptors (PRRs) to identify certain chemicals linked to pathogens (PAMPs) when they enter. PAMP-triggered immunity (PTI) is the defensive mechanism that is activated.^130^ Effector-Triggered Immunity (ETI) – To inhibit PTI, several pathogens use effector proteins (Figure 3). Plants have responded by developing resistance (R) genes that can identify these effectors and trigger a more robust defensive response, which frequently entails the activation of defense-related genes and localized cell death (hypersensitive response).^131^

#### 5.4.2. Insect feeding and damage

Mechanical Damage: Plant tissues are physically harmed by phytopathogens and herbivores, which trigger genes that respond to wounds that result. Signaling chemicals such as jasmonic acid (JA) are produced by these genes.^132^ Jasmonic acid signaling (JA) is essential for controlling the expression of genes that protect against herbivory and the action of other spoilage organisms. This involves the synthesis of secondary metabolites (such as phenolics and alkaloids) that strengthen the plant’s defenses against herbivores.^133,134^

#### 5.4.3. Cross-talk between pathways

Integrative Responses: Signaling pathways (such as JA and salicylic acid [SA]) may interact when a plant is attacked by both insects and diseases at the same time. Depending on the perceived threat, this cross-talk enables plants to prioritize their defense responses, which may result in the overexpression or downregulation of certain genes.^135^ Systemic Acquired Resistance (SAR) – Plants can develop a systemic response following initial infection or injury, strengthening defenses in distant tissues as well as at the infection site. PR genes and other defense-related genes are expressed in this way.^136^

#### 5.4.4. Epigenetic modifications

Extended contact with pathogens or herbivores can result in epigenetic modifications, such as histone modification and DNA methylation, which affect the patterns of gene expression in a heritable way. Without altering the DNA sequence itself, this can prepare progeny for increased resistance.^137^

#### 5.4.5. Metabolic alterations

Modifications in gene expression may result in the synthesis of defense-related substances such as phytoalexins, which directly prevent the development of pathogens or discourage the feeding of insects.^138^

### 5.5. Gene expression

The preparation for expressing genes takes place in plants in response to insects with a huge number of genes undergoing up and down-regulation. The existence of whole-genome sequences in genomics and transcriptomics revealed sequence tags and microarrays that ensure better cognition of alterations that are observed in gene expression in response to the attacks from insects.^139^ DNA microarray supplies an accurate and closer observation of gene-expression methods and signaling arbitrated by plant signals and insect elicitors which showed its exceptional abilities to observe simultaneous gene expressions.^140^ The introduction of next-generation sequencing (NGS) techniques, and alternative methods including reduced represented sequencing, RNA-sequencing, and RAD-sequencing will be introduced as substitutes for microarrays for directly measuring gene expression. The expression of genes has been revolutionized by the expression quantitative trait loci (eQTL). The eQTL mapping possesses the benefit of addressing thousands of characters in a specific period and has been utilized in some crops like Arabidopsis,^141^ potato and tomato,^142^ rice,^143^ melon,^144^ and maize.^145^

Alteration in the profiles and expression of genes after herbivory has revealed a significant percentage of plant defense. The level of gene expression has likewise been utilized to analyze the variation in transcriptional profiles of genotypic changes. The gene expression is induced by Lepidopterans that take part in the glucosinolate metabolism, signal transduction, detoxification, and cell survival.^146^ On the other hand, gene expression including oxidative stress, glucosinolate synthesis, calcium-dependent signaling, and cell wall modification is controlled by aphids.^147^ Various attackers undergo unusual effects on plants based on their nutrition habits and attack on plants as observed in *Arabidopsis thaliana* transcriptional changes in response to their attack by *Bemisia tabaci* and *Myzus persicae*.^22^ The collection of some technologies including genetics, and various tools used in genetics like microarrays, proteomics, deep sequencing, and transcriptional profiling tools via mass spectrometry will improve our knowledge of plant defense molecular mechanisms against insects.^148^

### 5.6. Detoxification and modification response against insect attack

Insects can detoxify toxic metabolites produced by plants attacked with the aid of cytochrome P450 monooxygenases and glutathione 5-transferases. The enzyme is produced by the insects when the insects come in contact with the toxic metabolites stimulated by the plants like xanthotoxin in corn earworms.^149^ Detoxification enzymes take part in biological processes by being involved in the target region to modify toxic substances produced by the insect.^150^ The biochemical characterization of insect resistance as reported in various studies of chemical derivatives like pesticides and insecticides is associated with specific insensitive regions and detoxification of pesticides via metabolic enzymes including Glutathione S-transferases (GSTs), Acetylcholinesterase (AChE), and Carboxylesterase (CarE).^151^ These enzymes carry out major functions in xenobiotic detoxification. In the process of pesticide detoxification, the enzymes are regarded as biomarkers to explain the rate of resistance, susceptibility, and tolerance found in the body of the organisms. AChE is a major enzyme for regulating the hydrolysis of neurotransmitters produced by the release of acetylcholine (ACh) present in the organism’s nervous system.^152^ CarE is another essential detoxifying enzyme that performs pesticide resistance. GST are affected enzyme during the process of xenobiotics detoxification.^152^ Detoxifying enzymes protect insects from the injurious potential of pesticides on their body. These enzymes also assist in lysis of biologically active substances, hormones, and pheromones.^153^ So, changes in the potential of detoxification enzymes reveal resistance against insecticides used on insect and their adaptation to the plant and their growth during metamorphosis. Detoxification processes are essential to plant defense during their interactions with herbivores and or phytopathogens. Numerous physiological and biochemical mechanisms have been developed by plants to fend off threats and detoxify toxic chemicals. Here are a few important detoxification mechanisms:

#### 5.6.1. Enzymatic detoxification of Phytotoxins

Cytochrome P450 Monooxygenases (CYPs): The enzymes known as monooxygenases (CYPs) oxidize harmful substances, aiding in the detoxification of pathogen and herbivore poisons. Secondary metabolites from plants can be hydroxylated by CYPs to become less poisonous and more hydrophilic forms. However, glutathione-S-transferases (GSTs) via conjugating glutathione to harmful substances, increase their solubility and facilitate their excretion or compartmentalization. This group of enzymes is essential for detoxifying secondary metabolites and reactive oxygen species (ROS) generated by pathogens and herbivores.

#### 5.6.2. Stimulation of secondary metabolite

Alkaloids, terpenoids, and phenolics – These substances are poisonous to phytopathogens and herbivores.^154^ For instance, the breakdown of alkaloids like nicotine and glucosinolates in Brassicaceae results in volatile chemicals that discourage herbivores and prevent the establishment of pathogens.^155^ Certain plants react to pathogen attacks by producing antimicrobial substances like phytoalexins. These substances impede or stop the spread of pathogens by interfering with their metabolism and cellular integrity.

#### 5.6.3. Reactive oxygen species (ROS)

When attacked by pathogens or herbivores, plants quickly release reactive oxygen species (ROS). To prevent self-harm, they must detoxify ROS as well. ROS are neutralized by enzymes such as ascorbate peroxidase (APX), catalase (CAT), and superoxide dismutase (SOD). Additionally, ROS can function as signaling molecules that set off other defensive reactions, such as the activation of genes that promote detoxification and protection.^156^

#### 5.6.4. Compartmentalization of toxins and cell wall fortification

Plants store harmful substances in the vacuole, a secure area away from the cellular machinery. For instance, when herbivores damage cyanogenic glycosides, which are kept in vacuoles in an inactive state, they release hydrogen cyanide.^37^ Before being transferred to different tissues or compartments, several toxins undergo modifications in the endoplasmic reticulum (ER) to lessen their toxicity. By depositing lignin, callose, and suberin, plants strengthen their cell walls, creating barriers that prevent herbivores and pathogens from penetrating.^157^ By limiting tissue damage to specific regions, this reaction not only lessens pathogen access but also lowers the spread of poisons.

## 6. Conclusion

For protection from insect attacks and phytopathogens, plants utilize biochemical and molecular defenses. While indirect defenses, such as the release of volatile organic compounds (VOCs), draw herbivore predators, direct defenses entail the production of harmful chemicals at wound sites. Plant defenses have been strengthened by biotechnology advancements, particularly with genetically modified crops (like Bt maize) that have *Bacillus thuringiensis* genes for insecticidal effects. In addition to secondary metabolites that suppress diseases and repel insects, physical barriers such as trichomes, thorns, and waxy cuticles also aid in discouraging herbivores. Plants engage defense responses through signaling pathways involving salicylic acid and jasmonic acid, which can also be transferred to progeny through transgenerational-induced resistance (TIR). To promote sustainable agriculture, further study is required on TIR, signaling pathways, and epigenetics despite these advancements.
